# Differences in healthcare use and mortality in older adults during the COVID-19 pandemic: Exploring long-term care users' vulnerability

**DOI:** 10.1016/j.heliyon.2024.e34840

**Published:** 2024-07-18

**Authors:** Isabel Aguilar-Palacio, Lina Maldonado, Sara Malo, Sara Castel-Feced, Alberto Cebollada, Alejandra Aguilar-Latorre, M José Rabanaque

**Affiliations:** a Department of Preventive Medicine and Public Health, University of Zaragoza, Zaragoza, Spain; b Grupo de Investigación en Servicios Sanitarios de Aragón (GRISSA), Fundación Instituto de Investigación Sanitaria de Aragón (IIS Aragón), Zaragoza, Spain; c Research Network on Chronicity, Primary Care and Health Promotion (RICAPPS), Carlos III Health Institute (ISCIII), Madrid, Spain; d Department of Applied Economics, University of Zaragoza, Zaragoza, Spain; e Biocomputing Unit, Institute for Health Sciences in Aragon (IACS), Zaragoza, Spain; f Aragonese Primary Care Research Group (GAIAP), Institute for Health Research Aragón (IIS Aragón), Zaragoza, Spain

**Keywords:** Health care utilization, Health care inequalities, COVID-19, Older adults, Nursing homes

## Abstract

**Background:**

The objective of our study is to analyze the health care received by older adults with COVID-19 according to their place of residence (whether or not they live in a long-term care [LTC] facility) and to find out the effect of health care on mortality.

**Methods:**

Retrospective cohort study based in Aragón (Spain) from March 2020 to March 2021 in patients aged 65 years or older with a confirmed COVID-19 infection. The population was classified according to their place of residence (living in a LTC or not). A propensity score was used to match individuals by sex and age. The effect of living in a LTC facility on healthcare delivery and mortality was conducted using adjusted multivariate models. Varimp was used to estimate the best predictors of mortality for both groups.

**Results:**

Healthcare services utilization varied depending on whether the patients lived in a LTC facility or not. The time to diagnosis was shorter in institutionalized patients, but the time to hospital admission was longer. Length of hospital stays, risk of ICU admission and 30-day mortality were also different and remained statistically significant in the adjusted models. The variables that were more important in the association between healthcare utilization and mortality were those associated with greater severity of COVID-19.

**Conclusions:**

There were differences in health care for older adults diagnosed with COVID-19 according to their place of residence. There is a need to strengthen collaboration between professionals in LTC centers and health services to provide equitable health care.

## 1 Background

The impact of pandemics on the health of populations is uneven, and COVID-19 is no exception. Two years after the start of the pandemic, studies have highlighted the differential risk of infection with COVID-19 according to gender, ethnicity, socio-economic position or residential area, showing a higher risk of infection in vulnerable groups [[1], [2], [3]]. Thus, as many authors have pointed out, COVID-19 has killed unequally, it has been experienced unequally and is also impoverishing societies unequally [4].

Older adults have been identified as one of the most vulnerable groups in studies of pandemic risk. Older people infected with SARS-CoV-2 are at higher risk of death and disability [5]. The presence of comorbid conditions [6], age-related decline, and immune dysregulation [7], among other factors, can explain, at least in part, the higher morbidity and mortality in this group. Social vulnerability also plays a role in this age group, exacerbating existing inequities [8].

Among older adults, people living in long-term care (LTC) facilities have presented the highest mortality rates of COVID-19 [9,10]. In Spain, an excess of 26,448 deaths have been estimated to occur in LTC facilities between March 2020 and May 2021. This represents about 10 % of the total number of people in nursing homes [11]. Various theories have attempted to explain this high mortality. Some theories have related this excess mortality to a higher presence of comorbidities and frailty of patients in LTC facilities [[12], [13], [14]]. However, other theories suggest that these differences may be related to staff-related factors such as inadequate training [15] or staff shortages [16], and facility characteristics, such as physical space or occupancy [17]. Finally, poor organizational and policy responses may have resulted in poorer care for these patients [18,19]. All these studies have addressed individual and organizational aspects to explain the highest mortality in people residing in LTC facilities. However, in other health crises and in other vulnerable populations [13], barriers to healthcare access and provision have also shown its impact. For this reason, it is crucial to assess the health care of older people living in nursing homes.

The aim of our study is twofold. Firstly, to compare the health care attention received by elderly patients with COVID-19 living in LCT facilities with that received by non-institutionalized people, in order to explore the existence of healthcare differences. Secondly, to understand the effect of healthcare attention on mortality in these patients. By fulfilling these objectives, the aim is to increase knowledge of the causes that may have led to this excess mortality in LTC facilities, analysing the role of the healthcare system. The ultimate aim is to improve health care in the face of future health crises in this group.

## 2 Methods

### 2.1 Design and study population

Retrospective cohort study. The setting of this study is Aragón, an Autonomous Community in north-eastern Spain (1.3 million inhabitants).

The data for the analyses were sourced from the Aragón-COVID19 cohort, which compiles information on all individuals tested for COVID-19 in Aragón. This cohort's data is derived from administrative health records and the electronic health records of the Aragón Health Service. In Spain, the healthcare system is predominantly tax-funded and operates on the principles of universal access, equity, free access, and fair financing [20].

The Aragón-COVID19 cohort includes individuals tested for COVID-19 due to either presenting symptoms consistent with the virus or having close contact with a confirmed case. COVID-19 cases were verified using polymerase chain reaction (PCR) or antigen tests. For this study, we analysed data from March 9, 2020, the first epidemiological week with reported COVID-19 cases in Aragón, to March 14, 2021, marking the end of the fourth wave. By this date, there were 103,281 confirmed COVID-19 cases. From this dataset, we focused on individuals aged 65 years or older with confirmed COVID-19 infections.

### 2.2 Data definition

We used sociodemographic, clinical and healthcare utilization data. Sociodemographic characteristics analysed were, in the first instance, sex and age. We also took into account socioeconomic level, calculated on the basis of the level of pharmacy copayment and social security benefits received. It depends on the type of user of the Aragón health service. The combination of these two variables resulted in 5 mutually exclusive categories: people with a contributory pension < €18,000 per year; people with a contributory pension ≥ €18,000 per year; people belonging to the mutual insurance scheme for civil servants; people with free medicines (people with minimum integration income or who no longer receive the unemployment benefit); and other situations not previously considered. Finally, we classified the area of residence into urban (those areas that concentrate at least 80 % of the population in their municipalities) or rural, according to the Aragon Government criteria [21].

We used the morbidity-adjusted groups (GMA) [22] in order to know patients’ clinical status. GMA takes into account medical diagnoses available in primary care and hospitalization databases. We linked each patient to their GMA information from January 2020, in order to know the health status prior to the COVID-19. We considered the presence of a diagnosis of Diabetes Mellitus, hypertension, stroke, ischemic heart disease, heart failure, chronic obstructive pulmonary disease (COPD), chronic kidney disease, depression, or dementia. We selected these clinical diagnoses due to their high prevalence in old people. Finally, we used also GMA weight to estimate the complexity, obtained from the aggregation of the patient's different diagnoses.

Information about the beginning of COVID-19 symptoms and diagnosis was obtained from the information system of primary healthcare. From CMBD of Aragón, we obtained information about hospital and intensive care unit (ICU) admission and mortality.

### 2.3 Statistical analyses

First of all, we analysed the association between living in an LTC facility with sex and age Mann–Whitney and Chi-square tests and standardized mean differences (SMD), as sex and age could influence the outcomes studied and the approach chosen. Those variables had a p-value of <0.2 and/or standardized differences >0.1. Because of this, sex and age were used to build a propensity score to avoid selection bias. Propensity analysis is conducted to adjust the selection bias between two groups.We applied a propensity score matching (PSM) through the nearest-neighbor method with and without replacement. Criteria: distance = glm’: This specifies the method for calculating distances between units. ‘glm’ indicates the use of generalized linear models; ratio = 1: This sets the ratio of treated to control units in the matched sample. In this case, it's set to 1, meaning a 1:1 matching; estimand = ‘ATT’: This specifies the treatment effect to be estimated. ‘ATT’ stands for Average Treatment Effect on the Treated; method = “nearest”: This specifies the matching method. ‘nearest’ is a nearest-neighbor matching method, where each treated unit is matched to the nearest control unit based on the estimated propensity score; caliper = 0.1: This specifies the caliper width, which restricts the set of eligible matches to those within a specified distance (caliper) of the estimated propensity score. In this case, the caliper is set to 0.1. Covariate balance was assessed by SMD, before and after weighting or matching considering SMD <0.1 or 0.05 as good.

After matching, we described socioeconomic and morbidity characteristics, as well as healthcare delivery for each group. We evaluated, in patients with symptoms, the time from onset of COVID-19 symptoms to diagnosis, with a range of −7 to 15 days. Time from the COVID-19 diagnosis to hospital admission (−15 to 30 days) was also calculated. Hospital admission (yes/no) and ICU admission (yes/no) in this hospitalization were obtained. As we do not have the hospitalization diagnosis, we considered a hospitalization to be related to COVID-19 if it occurred between −15 and 30 days of the COVID-19 diagnosis. The length of hospital stay and the length of ICU stay was calculated. Finally, we evaluated mortality from any cause at 7, 30, and 90 days after COVID-19 diagnosis and the effect of health care on mortality.

To analyze the effect of living in an LTC facility on healthcare utilization we performed multivariate analyses. To assess the relationship between place of residence and time from symptom onset to confirmation and from diagnoses to confirmation, multivariate linear regression analyses were conducted. Logistic regression models were used to examine the influence of residence location on hospital and ICU admissions. For the length of hospital stay and length of ICU stay, Poisson regression models were made. All models were adjusted by socioeconomic status, type of residence area (rural or urban), pandemic wave, and the presence of comorbidities (Diabetes Mellitus, hypertension, stroke, ischemic heart disease, heart failure, COPD, chronic kidney disease, depression, or dementia). Finally, we conducted logistic regression models for mortality including healthcare delivery indicators. These models were stratified by place of residence (LTC facility or not). We calculated the best predictors of mortality for both non-institutionalized and institutionalized patients using Varimp. All the analyses were conducted using R 4.1.3 (2022-03-10).

This study was approved by The Clinical Research Ethics Committee of Aragón (CEICA) (protocol code: PI20/184).

## 3 Results

21,235 people over 64 years of age had a confirmed COVID-19 diagnosis in Aragón for the period considered. Of this population, 4360 people (20.5 %) were living in an LTC facility. Information about this population by place of residence is available in Supplemental Material Table 1.

From this population, we obtained a sex and age matched sample of 8648 COVID-19 patients, composed of 65.5 % of women with a mean age of 86.5 years old. When stratifying the sample by place of residence, differences were observed by socioeconomic level, type of basic healthcare area of residence, complexity, stroke, depression, and dementia. Older people residing in LTC facilities had lower socioeconomic status and lived in rural basic healthcare areas with higher frequency than those non-institutionalized. Regarding morbidities, institutionalized patients presented higher complexity, and had stroke, depression, and dementia with more frequency than the other group (Table 1).

**Table 1:** Population description according to institutionalization.

	Global	Non- institutionalized	Institutionalized	p
	(N = 8648)	(N = 4324)	(N = 4324)	
Age^a^	86.5 (7.26) [83:91]	86.5 (7.26) [83:91]	86.5 (7.26) [83:91]	0.978
Sex^b^				0.667
Male	2986 (34.5 %)	1503 (34.8 %)	1483 (34.3 %)	
Female	5662 (65.5 %)	2821 (65.2 %)	2841 (65.7 %)	
Socioeconomic level^b^				<0.001*
Mutualist	288 (3.33 %)	137 (3.17 %)	151 (3.49 %)	
Pensioner <18,000€/year	6534 (75.6 %)	3144 (72.7 %)	3390 (78.4 %)	
Pensioner ≥18,000€/year	1357 (15.7 %)	782 (18.1 %)	575 (13.3 %)	
Free medicines	373 (4.31 %)	202 (4.67 %)	171 (3.95 %)	
Other	96 (1.11 %)	59 (1.36 %)	37 (0.86 %)	
Type of Basic Healthcare Area^b^				0.005*
Rural	2748 (32.0 %)	1305 (30.6 %)	1443 (33.4 %)	
Urban	5839 (68.0 %)	2962 (69.4 %)	2877 (66.6 %)	
Complexity^a^	11.6 (5.98) [7.34; 15.10]	11.0 (5.88) [6.85; 14.49]	12.1 (6.03) [7.92; 15.68]	<0.001*
Morbidity^b^				
Diabetes Mellitus	2164 (25.9 %)	1059 (25.4 %)	1105 (26.4 %)	0.284
Heart failure	1060 (12.7 %)	524 (12.6 %)	536 (12.8 %)	0.743
Ischemic heart disease	854 (10.2 %)	431 (10.3 %)	423 (10.1 %)	0.778
Stroke	1004 (12.0 %)	394 (9.44 %)	610 (14.6 %)	<0.001*
Hypertension	6025 (72.1 %)	3009 (72.1 %)	3016 (72.1 %)	0.995
COPD	814 (9.74 %)	399 (9.56 %)	415 (9.92 %)	0.600
Chronic kidney disease	2281 (27.3 %)	1120 (26.8 %)	1161 (27.8 %)	0.353
Depression	2097 (25.1 %)	917 (22.0 %)	1180 (28.2 %)	<0.001*
Dementia	2034 (24.3 %)	642 (15.4 %)	1392 (33.3 %)	<0.001*

N, number; p, statistical significance; a, Mean (Standard Deviation) [interquartile range]; b, Number (percentage); COPD, chronic obstructive pulmonary disease. *Statistically significant results.

We analysed healthcare delivery by both groups considered. Time to diagnosis was lower in those living in an LTC facility than in no institutionalized (0.69 days vs. 2.36). On the contrary, the time to hospitalization was higher in institutionalized patients (3.41 days vs. 2.30). The probability of hospital admission was similar for both groups, but the length of hospital stay was significantly higher in institutionalized patients (p = 0.003). We also observed differences in the probability of ICU admission, with higher admission in the no-institutionalized group. Mortality was higher in people living in an LTC facility at 30 and 90 days of COVID-19 diagnosis, but no differences were observed for mortality at 7 days (Table 2).

**Table 2:** Healthcare delivery and mortality according to institutionalization. Bivariate analyses.

	Non- institutionalized	Institutionalized	p
	(N = 4324)	(N = 4324)	
Time to diagnosis (days)^a^	2.36 (3.59)	0.69 (3.39)	<0.001*
Time to hospital admission (days)^a^	2.30 (5.15)	3.41 (6.20)	<0.001*
Hospital admission^b^	1766 (40.8 %)	1739 (40.2 %)	0.569
Length of hospital stay (days)^a^	14.3 (14.5)	16.1 (18.7)	0.003*
ICU admission^b^	50 (1.16 %)	15 (0.35 %)	<0.001*
Length of ICU stay (days)^a^	19.5 (15.2)	15.6 (15.5)	0.243
Mortality at 7 days^b^	328 (7.59 %)	325 (7.52 %)	0.935
Mortality at 30 days^b^	764 (17.7 %)	1025 (23.7 %)	<0.001*
Mortality at 90 days^b^	883 (20.4 %)	1316 (30.4 %)	<0.001*

a Mean (standard deviation).

B Number (%)*Statistically significant results.

To explore the effect of living in a LTC facility, univariate and multivariate models for each healthcare indicator were performed. After adjusting by socioeconomic level, type of zone, wave, and comorbidities, we observed that institutionalized people were diagnosed with COVID-19 1.66 days before than non-institutionalized (from 1.86 to 1.47 days before). Time to hospital admission was higher in people living in an LTC facility than non-institutionalized (1.10 days later; 95%CI 0.71–1.50 days). The multivariate model was not conducted for hospital admission as no differences were observed between institutionalized and non-institutionalized in the univariate model. The expected length of hospital stay in institutionalized patients was 1.15 days more than in non-institutionalized. ICU admission was lower in institutionalized people. Also, after adjusting by covariates, the expected length of ICU stay was 1.24 days more in institutionalized patients (95%CI 1.04–1.46).

There were differences in the risk of mortality at 30 and 90 days after adjusting for covariates. The risk of death was higher in those living in an LTC facility than in non-institutionalized. No differences were observed at 7 days (Table 3).

**Table 3:** Risk of old institutionalized people in relation to non-institutionalized for healthcare delivery and mortality. Univariate and multivariate models.

	Univariate model			Multivariate model		
	OR	p-value	95%CI	OR	p-value	95%CI
Time to diagnosis (days)^a^	−1.67	<0.001	−1.86–−1.48	−1.66	<0.001	−1.86–−1.47
Time to hospital admission (days)^a^	1.11	<0.001	0.73–1.49	1.10	<0.001	0.71–1.50
Hospital admission^b^	0.97	0.554	0.89–1.06	–		–
Length of hospital stay (days)^c^	1.13	<0.001	1.11–1.15	1.15	<0.001	1.13–1.17
ICU admission^b^	0.30	<0.001	0.16–0.52	0.36	0.001	0.19–0.65
Length of ICU stay (days)^c^	0.79	0.001	0.68–0.90	1.24	0.014	1.04–1.46
Mortality at 7 days^b^	0.99	0.903	0.84–1.16	–		–
Mortality at 30 days^b^	1.45	<0.001	1.30–1.61	1.36	<0.001	1.22–1.52
Mortality at 90 days^b^	1.70	<0.001	1.55–1.88	1.61	<0.001	1.45–1.79

OR: Odds ratio; 95%CI: 95 % Confidence interval; ^a^ Linear regression model ^b^ Logistic regression models ^c^ Poisson model; adjustment variables: socioeconomic level, type of zone, wave, Diabetes Mellitus, Heart failure Ischemic heart disease, Stroke, Hypertension, COPD, Chronic kidney disease, Depression and Dementia.

**Fig. 1:**
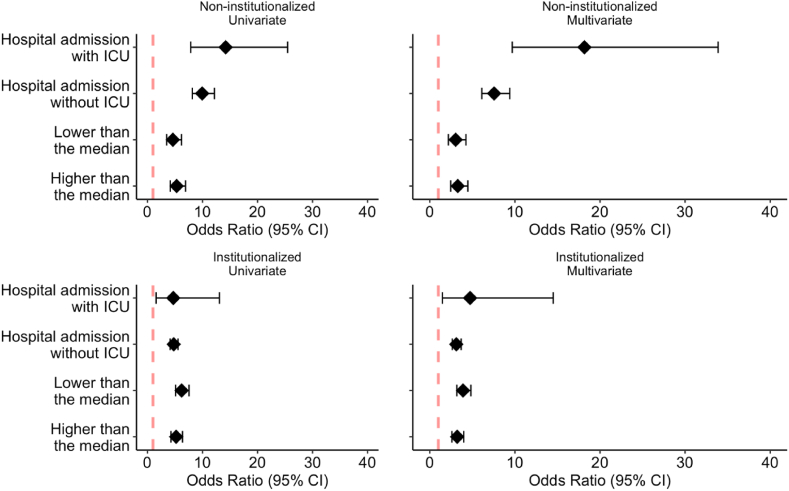
Healthcare delivery and its association with the risk of mortality at 30 days (left) and 90 days (right) stratified by place of residence.

We evaluated the effect of healthcare delivery on mortality for both institutionalized and non-institutionalized patients at 30 and 90 days (Fig. 1). The existence of a hospital stay increased the risk of dying both at 30 and 90 days compared with no hospital stay. This effect was higher in non-institutionalized people and showed no statistically significant differences between the categories with and without ICU stay. Regarding time from the beginning of symptoms to diagnosis, the existence of symptoms was a risk factor for dying, but no differences were observed when the time from symptoms to diagnosis was evaluated.

Reference values: no hospital admission; no symptoms. Model adjustment variables: socioeconomic level, type of zone, wave, Diabetes Mellitus, Heart failure, Ischemic heart disease, Stroke, Hypertension, COPD, Chronic kidney disease, Depression, and Dementia.

**Fig. 2:**
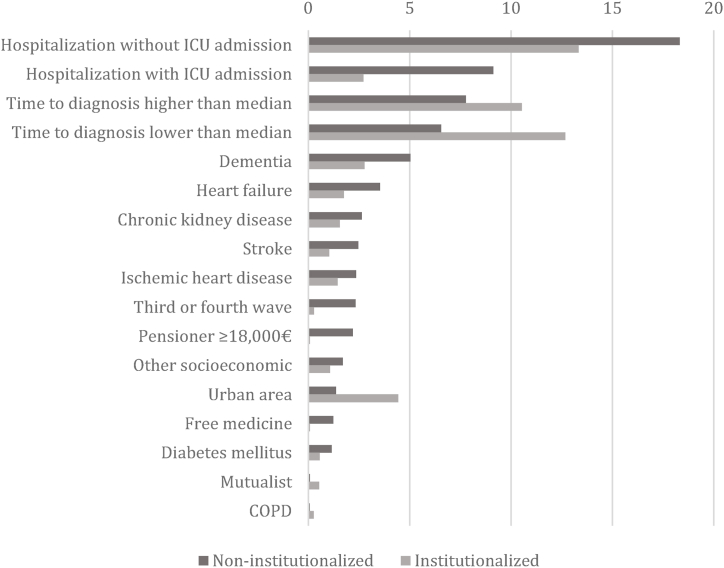
Predictors of mortality at 30 days. Varimp analyses.

Finally, we calculated the best predictors of mortality for both non-institutionalized and institutionalized patients. The most important factor for both groups was hospital admission without ICU admission, followed by hospital admission with ICU admission in the case of non-institutionalized patients. The time to diagnosis was also a relevant fact, especially in patients living in an LTC facility (reference category: no symptoms). Finally, the presence of the different comorbidities was more important for 30-day mortality in non-institutionalized than in institutionalized patients. (Fig. 2).

## 4 Discussion

In this study of people aged 64 and over with a COVID-19 diagnosis, we found that those residing in LTC facilities had a lower socioeconomic status and were more complex patients in terms of morbidity, with a higher incidence of stroke, depression and dementia, than non-institutionalized patients.

The use of health services related to the COVID-19 episode also differed according to whether the patients lived in an LTC facility or not. We found that the time to diagnosis was shorter in institutionalized than in non-institutionalized. In contrast, the time to hospital admission was longer in institutionalized patients. Differences were also observed in the lenght of hospital stay (longer in institutionalized patients), the risk of ICU admission (lower in institutionalized patients), and mortality at 30 days or more (higher in institutionalized patients). All these differences remained statistically significant after adjustment for socioeconomic status of the subject, the area of residence, the epidemic wave, and patient comorbidities. The length of stay in the ICU became significant in the multivariate analysis, with a longer duration in patients from LTC facilities. However, there were no differences between groups in the risk of hospital admission. When we considered the importance of the variables related to healthcare utilization on mortality, we observed that those that were significant were those associated with greater severity of the COVID case (presence of admission and symptoms), with the role of comorbidities being much smaller.

The time from the beginning of symptoms to diagnosis was shorter in the population residing in LTC facilities. This is probably associated with a better diagnostic strategy, given the high vulnerability of LTC patients and the known importance of minimizing testing delays in order to reduce COVID-19 transmission [23]. Also, in the first pandemic wave, diagnostic tests were available earlier in the institutional context.

The risk of hospital admission was similar for both groups when sociodemographic and morbidity variables were considered. Nonetheless, a longer time from diagnosis to hospital admission was observed for LTC patients. This fact has been described in other European studies [24]. In Spain, difficulties to hospital admission have also been identified in these patients [25]. There are some explanations for this delay. The existence of patients with a higher degree of deterioration in institutions, observed in a higher frequency of stroke and dementia, has been associated with a higher risk of mortality without hospital admission [12]. In these patients, the symptoms of COVID-19 infection are atypical and are usually related to worsening functional status [26,27]. This makes clinical decision-making more complex and can lead to a delay in care. Also, many LTC facilities have low staffing ratios, lack medical resources, and show poor coordination between social and health services, which hinders access to health resources [28].

The risk of death in people living in LTC facilities was higher than in the same age and sex population non-institutionalized. This result is in line with the literature [29,30]. This fact may be associated with greater severity in these cases. The existence of prior deterioration has been associated with a worse prognosis for COVID-19 [31]. This higher severity could also explain the longer time of hospital and ICU stay in this group. Despite the higher mortality observed in patients living in LTC facilities, their risk of ICU admission was lower. When ICUs became overwhelmed by COVID-19, critical care triage to prioritize patients was required [32]. A systematic review conducted to understand the prioritization criteria for ICU admission during periods of high demand concluded that the principle of utility was the most commonly used to triage resources [33].

Finally, regarding mortality predictors in both groups, we observed that those variables related with the severity of COVID-19 (as hospitalization with or without ICU admission) had more importance on mortality than previous comorbidities. This fact has also been described in other studies [34]. Although many authors have highlighted the importance of comorbidities in COVID-19 mortality [14,35], probably the advanced age of our population and the high frequency of comorbidities in both groups make these previous diseases less determinant of COVID-19 mortality in this study. Also, living in an urban area was a stronger predictor of mortality in people residing in LTC facilities than in non-institutionalized. These results are in line with other publications [36,37]. This could be related to factors associated with LTC facilities characteristics, such as private ownership [38], which may have led to excess risk in the urban environment.

This study has some limitations. To overcome the limitation of missing cause of hospital admission, a range of −15 to 30 days from hospital admission to COVID-19 was used. The cause of death was also not available, so we considered mortality at different times after the COVID-19 diagnosis. Some of the institutionalized patients could have been treated in one of the “COVID centers” set up in Aragón at the beginning of the pandemic, but this information was not recorded. With regard to the methods used, the use of matching techniques enabled us to manage the influence of sex and age. Nevertheless, this approach has hindered our ability to evaluate how both variables impact both healthcare utilization and mortality. Future studies investigating the impact of gender on healthcare, encompassing both biological factors and possible gender-related biases, along with the role of age in healthcare, should consider an alternative methodology. Finally, the limitations of observational studies based on real-world data, such as data quality and the presence of incomplete data, must be taken into account.

On the other hand, this study has several strengths. This study is based on a large European population. From this population, we selected all the individuals aged 64 years or more residing in an institution with a confirmed COVID-19 infection and compared them with a sample of non-institutionalized patients balanced by sex and age. We included data from administrative health data sources and electronic health records. Lastly, morbidities were derived from GMA, a data source that integrates diagnoses from both primary healthcare and hospital admissions, resulting in a highly sensitive classification.

## 5 Conclusions and recommendations

We observed that healthcare delivery in old patients with a COVID-19 diagnosis differed according to the place of residence, with differences in time to diagnosis, time to hospital admission, risk of ICU admission, and length of hospital and ICU stay. In order to provide equitable healthcare attention, regardless of whether individuals live in an institution or not, it is necessary to strengthen collaboration between LTC facilities professionals and health services, to improve coordination between levels [39]. In this sense, some proposals are the implementation of integrated care models that promote seamless care transitions and interdisciplinary collaboration. In this sense, establishing multidisciplinary teams can also help in addressing the needs of patients. Investment in qualified staff and resources is crucial, alongside providing comprehensive training and guidance on transitional care to ensure optimal support during changes in care settings [40]. Additionally, developing clear protocols and maintaining ongoing communication are essential for ensuring consistent and high-quality care [41]. Further strategies could involve leveraging technology, such as electronic health records and telehealth services, to enhance information sharing and provide remote consultations. Finally, establishing audits and feedback mechanisms to continually assess and improve the effectiveness of coordination efforts, ensuring that all stakeholders are aligned and responsive to the evolving needs of patients is crucial in order to ensure that all patients receive attention in a timely and appropriate manner.

It is also important to highlight that statistically significant results do not always imply clinically relevant results. For future studies along these lines, some methodological improvements should be implemented. We recommend considering the use of more advanced techniques for adjusting and validating propensity scores. In particular, the use of stricter calipers and bootstrapping methods to validate results may be beneficial. It is also important to improve data quality and integration by linking multiple data sources, such as hospitalization records, mortality records and public health data. By integrating these datasets, researchers can gain a more comprehensive view of patients' health status and care pathways. This would allow better identification of the causes of hospitalization and death. In addition, more complete data will allow a more thorough analysis of health care use and outcomes, leading to more accurate and reliable research results.

## Ethics approval and consent to participate

The research protocol of this study was approved by The Clinical Research Ethics Committee of Aragón (CEICA) (PI20/184). Consent to participate was not necessary because this was a secondary analysis study and data were pseudonymized.

## Data availability statement

Aragón-COVID19 cohort data has not been deposited into a publicly available repository. Data will be made available on request.

## CRediT authorship contribution statement

**Isabel Aguilar-Palacio:** Writing – review & editing, Writing – original draft, Supervision, Methodology, Funding acquisition, Conceptualization. **Lina Maldonado:** Writing – review & editing, Methodology, Data curation. **Sara Malo:** Writing – review & editing, Investigation. **Sara Castel-Feced:** Writing – review & editing, Methodology, Data curation. **Alberto Cebollada:** Visualization, Methodology, Formal analysis. **Alejandra Aguilar-Latorre:** Writing – review & editing, Investigation. **Ma José Rabanaque:** Writing – review & editing, Funding acquisition, Conceptualization.

## Declaration of competing interest

The authors declare the following financial interests/personal relationships which may be considered as potential competing interests:Isabel Aguilar reports financial support was provided by 10.13039/501100010067Government of Aragón. Isabel Aguilar reports financial support was provided by Research Network on Chronicity Primary Care and Prevention and Health Promotion. If there are other authors, they declare that they have no known competing financial interests or personal relationships that could have appeared to influence the work reported in this paper.
