# Supplementary material for: Differences in healthcare use and mortality in older adults during the COVID-19 pandemic: Exploring long-term care users' vulnerability
Source: Heliyon. 2024 Jul 18;10(14):e34840. doi: 10.1016/j.heliyon.2024.e34840 (PMC11324964; doi:10.1016/j.heliyon.2024.e34840)
Supplement: Multimedia component 1 [file mmc1.docx]

**SUPPLEMENTAL MATERIAL**

**Table 1. People over 64 years of age with a COVID-19 confirmed diagnosis in Aragon.**

|  | **Global** | | **Non-institutionalized** | **Institutionalized** | **p.overall** |
| --- | --- | --- | --- | --- | --- |
|  | ***N=21,325*** | | ***N=16,965*** | ***N=4,360*** |  |
| Age^a^ | 79.2 (9.33) | | 77.3 (8.83) | 86.6 (7.28) | 0.000* |
| Sex^b^ |  | |  |  | <0.001* |
| Male | 9387 (44.0%) | | 7869 (46.4%) | 1518 (34.8%) |  |
| Female | 11938 (56.0%) | | 9096 (53.6%) | 2842 (65.2%) |  |
| Socioeconomic level^b^ | |  |  |  | <0.001* |
| Mutualist | | 663 (3.11%) | 511 (3.01%) | 152 (3.49%) |  |
| Pensioner <18,000€/year | | 13189 (61.8%) | 9768 (57.6%) | 3421 (78.5%) |  |
| Pensioner ≥18,000€/year | | 5712 (26.8%) | 5133 (30.3%) | 579 (13.3%) |  |
| Free medicines | | 1022 (4.79%) | 851 (5.02%) | 171 (3.92%) |  |
| Other | | 739 (3.47%) | 702 (4.14%) | 37 (0.85%) |  |
| Type of Basic Healthcare Area^b^ | |  |  |  | <0.001* |
| Rural | | 6324 (29.9%) | 4872 (29.0%) | 1452 (33.3%) |  |
| Urban | | 14820 (70.1%) | 11916 (71.0%) | 2904 (66.7%) |  |
| Complexity^a^ | | 9.75 (5.86) | 9.13 (5.65) | 12.1 (6.03) | <0.001* |
| Morbidity^b^ | |  |  |  |  |
| Diabetes Mellitus | | 5139 (25.0%) | 4027 (24.7%) | 1112 (26.4%) | 0.025* |
| Heart failure | | 1645 (8.01%) | 1105 (6.77%) | 540 (12.8%) | <0.001* |
| Ischemic heart disease | | 1991 (9.69%) | 1561 (9.56%) | 430 (10.2%) | 0.229 |
| Stroke | | 1622 (7.90%) | 1007 (6.17%) | 615 (14.6%) | <0.001* |
| Hypertension | | 13220 (64.4%) | 10180 (62.4%) | 3040 (72.1%) | <0.001* |
| COPD | | 1945 (9.47%) | 1525 (9.34%) | 420 (9.96%) | 0.237 |
| Chronic kidney disease | | 4095 (19.9%) | 2922 (17.9%) | 1173 (27.8%) | <0.001* |
| Depression | | 4155 (20.2%) | 2968 (18.2%) | 1187 (28.1%) | <0.001* |
| Dementia | | 2573 (12.5%) | 1172 (7.18%) | 1401 (33.2%) | <0.001* |

N, number; p, statistical significance; a, Mean (Standard Deviation); b, Number (percentage); COPD, chronic obstructive pulmonary disease. *Statistically significant results.
